# The *Medicago sativa* gene index 1.2: a web-accessible gene expression atlas for investigating expression differences between *Medicago sativa* subspecies

**DOI:** 10.1186/s12864-015-1718-7

**Published:** 2015-07-07

**Authors:** Jamie A. O’Rourke, Fengli Fu, Bruna Bucciarelli, S. Sam Yang, Deborah A. Samac, JoAnn F. S. Lamb, Maria J. Monteros, Michelle A. Graham, John W. Gronwald, Nick Krom, Jun Li, Xinbin Dai, Patrick X. Zhao, Carroll P. Vance

**Affiliations:** USDA-ARS, Corn Insects and Crop Genetics Research Unit, Ames, IA 50011 USA; Department of Agronomy and Plant Genetics, University of Minnesota, St. Paul, MN 55108 USA; USDA-ARS-Plant Science Research Unit, St. Paul, MN 55108 USA; Samuel Roberts Noble Foundation, Ardmore, OK 73401 USA; Present Address: Monsanto Company, Molecular Breeding Technology, Chesterfield, MO 63167 USA

**Keywords:** *Medicago sativa*, Illumina, RNA-seq, Gene expression atlas, Cysteine cluster protein, Nodule-specific cysteine-rich peptide

## Abstract

**Background:**

Alfalfa (*Medicago sativa* L.) is the primary forage legume crop species in the United States and plays essential economic and ecological roles in agricultural systems across the country. Modern alfalfa is the result of hybridization between tetraploid *M. sativa* ssp. *sativa* and *M. sativa* ssp. *falcata*. Due to its large and complex genome, there are few genomic resources available for alfalfa improvement.

**Results:**

A *de novo* transcriptome assembly from two alfalfa subspecies, *M. sativa* ssp. *sativa* (B47) and *M. sativa* ssp. *falcata* (F56) was developed using Illumina RNA-seq technology. Transcripts from roots, nitrogen-fixing root nodules, leaves, flowers, elongating stem internodes, and post-elongation stem internodes were assembled into the *Medicago sativa* Gene Index 1.2 (MSGI 1.2) representing 112,626 unique transcript sequences. Nodule-specific and transcripts involved in cell wall biosynthesis were identified. Statistical analyses identified 20,447 transcripts differentially expressed between the two subspecies. Pair-wise comparisons of each tissue combination identified 58,932 sequences differentially expressed in B47 and 69,143 sequences differentially expressed in F56. Comparing transcript abundance in floral tissues of B47 and F56 identified expression differences in sequences involved in anthocyanin and carotenoid synthesis, which determine flower pigmentation. Single nucleotide polymorphisms (SNPs) unique to each *M. sativa* subspecies (110,241) were identified.

**Conclusions:**

The *Medicago sativa* Gene Index 1.2 increases the expressed sequence data available for alfalfa by ninefold and can be expanded as additional experiments are performed. The MSGI 1.2 transcriptome sequences, annotations, expression profiles, and SNPs were assembled into the Alfalfa Gene Index and Expression Database (AGED) at http://plantgrn.noble.org/AGED/, a publicly available genomic resource for alfalfa improvement and legume research.

**Electronic supplementary material:**

The online version of this article (doi:10.1186/s12864-015-1718-7) contains supplementary material, which is available to authorized users.

## Background

Alfalfa is the most widely cultivated forage legume, with a global production area of 11–12 million ha [1]. In the United States, alfalfa is the fourth most widely produced crop [2], contributing more than $10 billion annually to the U.S. farm economy [3, 4]. Alfalfa produces high dry matter yields; 18–23 Mg/ha in the irrigated western U.S. and 7–14 Mg/ha in rain fed areas of the Eastern and Midwestern U.S. The crop is primarily used as feed in dairy cow, sheep, and beef production systems as dried hay, haylage, and for grazing. It is an excellent source of crude protein, vitamins, minerals, and the dietary fiber needed to maintain rumen health. Moreover, alfalfa contributes to the financial security of farmers by providing soil nitrogen (N) for subsequent crops in a rotation system due to its symbiotic N_2_ fixation capacity. Additionally, because alfalfa is a perennial crop, it provides numerous agro-ecological advantages including reduced soil erosion, improved soil carbon sequestration, and increased capture of nutrients from annual cropping fields to protect surface and ground water resources.

**Fig. 1:**
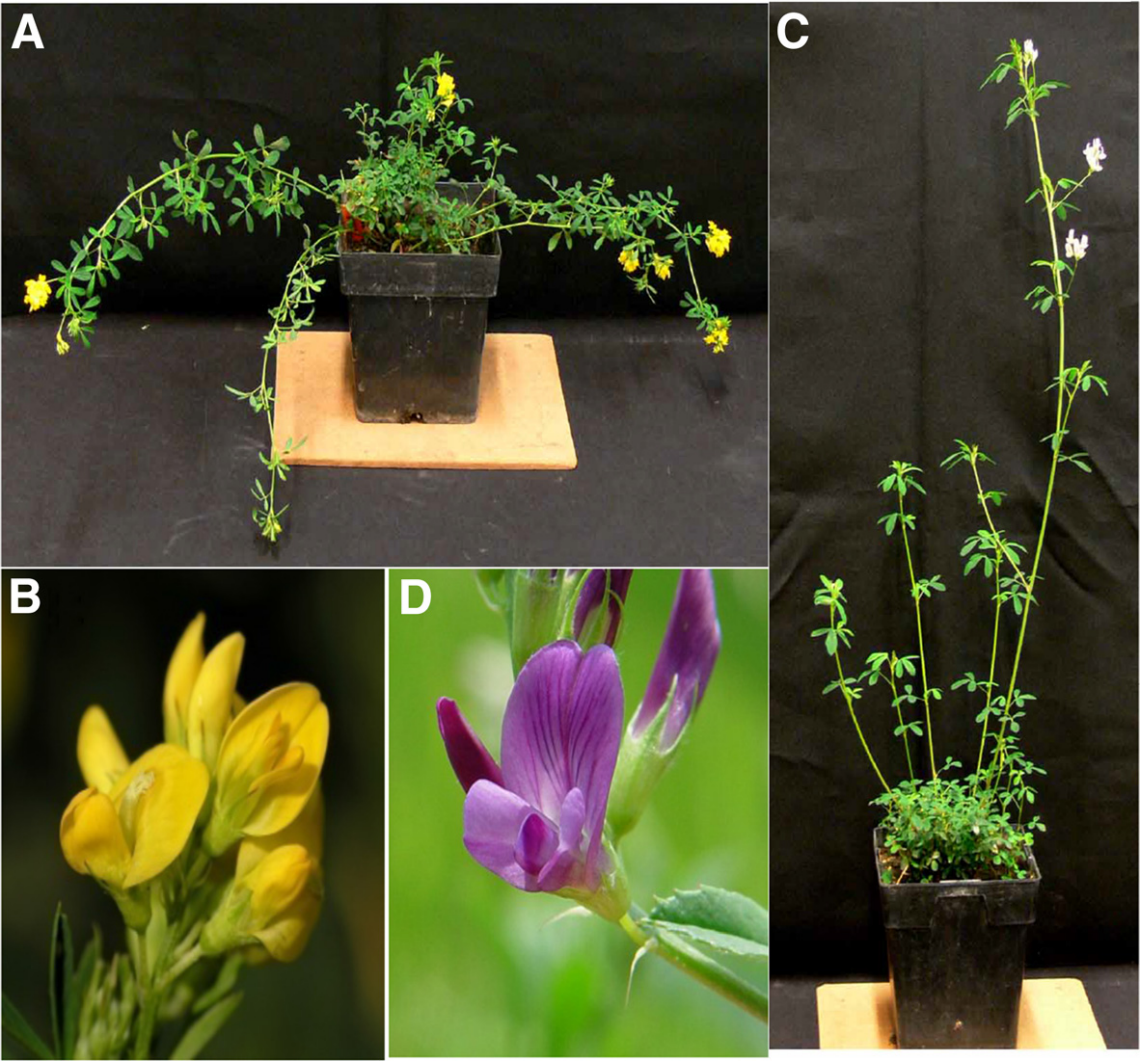
Phenotypes of *Medicago sativa* ssp. *falcata* and *Medicago sativa* ssp. *sativa*. **a** Recumbent stem architecture and (**b**) flower raceme of *Medicago sativa* ssp. *falcata* (F56). **c** Upright stem architecture and (**d**) flower raceme of *Medicago sativa* ssp. *sativa* (B47)

Most modern alfalfa cultivars grown in the U.S. are a result of introgression of *Medicago sativa* ssp. *falcata* and *M. sativa* ssp. *sativa*. The two subspecies readily hybridize although they have distinct phenotypes and geographic origins [5]. *M. sativa* ssp. *falcata* originated in central Asia and is characterized by orange-yellow flowers (Fig. 1a and b), straight to sickle-shaped seedpods, broad crowns, creeping-root habit, and extreme winter hardiness. Both diploid and autotetraploid accessions occur naturally. *M. sativa* ssp. *sativa* is an autotetraploid that originated in the Near East, with Iran as the geographic center of origin. *M. sativa* ssp. *sativa* has violet or lavender colored flowers (Fig. 1c and d), coiled seed pods, and is adapted to temperate regions. Both subspecies suffer from severe inbreeding depression when self-pollinated and are therefore bred as cross-pollinated synthetic cultivars. For this study, clones of one individual from each subspecies, *M. sativa* ssp. *sativa* (B47) and *M. sativa* ssp. *falcata* (F56), were selected for analysis. These lines exhibited superior performance when used as female parents in experiments to evaluate *M. sativa* ssp. *falcata* x *M. sativa* ssp. *sativa* semi-hybrids for enhancing forage yield (Lamb, unpublished).

Yield improvement in forage crops during the past century has lagged behind that of annual grain crops [6]. As an outcrossing tetraploid, genetic analysis of alfalfa is particularly difficult. Despite studies using 454 sequencing to identify SNPs [7], the development of an alfalfa SNP array [8], and the use of genotype by sequencing to develop an alfalfa linkage map [9] there is an overall paucity of genetic information and genomic resources that can be readily utilized by alfalfa breeders for alfalfa improvement. Gene expression atlases have been produced for a number of plants including *Arabidopsis thaliana* (Arabidopsis) [10], *Oryza sativa* (rice) [11], *Glycine max* (soybean) [12, 13], *Phaseolus vulgaris* (common bean) [14], and *Medicago truncatula* [15]. These have proven invaluable tools for understanding plant gene expression due to genome duplication, response to diverse environmental conditions, plant development, and pest and pathogen interactions [16–21]. Due to the close genetic relationship between *M. truncatula* and *M. sativa*, researchers have utilized the *M. truncatula* Affymetrix GeneChip to measure gene expression in homologous genes [22]. However, the genetic complexity of *M. sativa* severely limits this approach. Microarray technology is also constrained by prior knowledge of gene sequences, limiting the patterns of gene expression to a subset of the total transcriptional activity of an organism. As a result, microarrays provide only a fragmented picture of transcript accumulation patterns.

Next-generation sequencing has facilitated the development of transcriptome sequences prior to genome sequencing in several legume crop species including lentil [23], lupin [24], pea [25], pigeonpea [26], and red clover [27]. RNA-seq has been used for gene annotation, expression analysis, and SNP discovery. This methodology has also proven useful for discovery of novel transcripts (coding and non-coding) and identification of alternative splice variants. The Illumina RNA-seq platform allows for transcript identification and measurement of transcript abundance. It also has the advantage of higher sensitivity and greater dynamic range of expression than microarray-based technologies. Several Illumina-based RNA-seq studies have been performed in alfalfa though transcriptome analyses were limited to stems [28, 29], roots [30], shoots and roots [31], or were confined to a single cultivar [32].

The objectives of this research were to expand the available transcriptome data and develop an expression atlas for alfalfa that is accessible using a web-based interface for gene discovery and identification of molecular markers for alfalfa improvement. The atlas is based on a *de novo* transcriptome assembly (MSGI 1.2) for *M. sativa* ssp*. sativa* (B47) and *M. sativa* ssp. *falcata* (F56) using samples from roots, nitrogen-fixing root nodules, leaves, flowers, elongating stem internodes, and post-elongation stem internodes. The transcriptome assembly increases the expressed sequence data available for alfalfa by more than ninefold. In particular, the atlas provides the first transcriptome analysis from alfalfa root nodules. The alfalfa gene atlas data should prove useful in identifying genes and for delimiting intron and exon boundaries from genomic sequence. Here, we report transcripts differentially expressed between the two subspecies and tissue samples, and single nucleotide polymorphisms (SNPs) that differ between B47 and F56. We provide specific examples of the utility of the expression atlas for candidate gene identification. Comparing transcript abundance we identified specific expression differences for sequences potentially involved in cold tolerance, sequences in the anthocyanin and carotenoid synthesis pathways involved in yellow and purple flower pigmentation, cell wall related sequences differentially expressed between the two subspecies, and nodule-specific sequences unique to alfalfa. The entirety of the dataset has been assembled into the Alfalfa Gene Index and Expression Database (AGED), which is publicly available for download and exploration at http://plantgrn.noble.org/AGED/.

## Results and discussion

### *de novo* transcriptome assembly

**Fig. 2:**
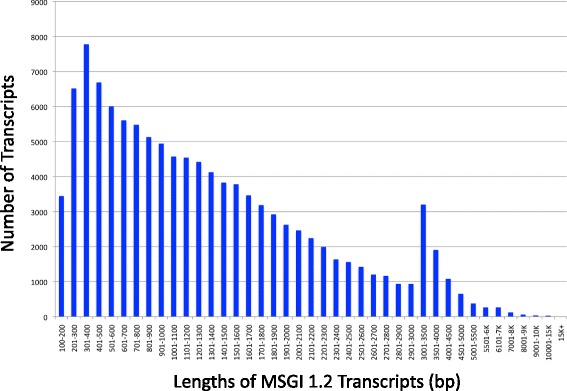
Distribution of transcript lengths in the *Medicago sativa* Gene Index 1.2. The *de novo* transcriptome was built from 76-bp single-end read sequences from three biological replicates of six tissues (roots, nodules, post-elongation stem internodes, elongating stem internodes, leaves, and flowers) isolated from *Medicago sativa* ssp. *falcata* (F56) and *M. sativa* ssp. *sativa* (B47) using Velvet/Oases, cd-HIT, and CAP3. The *Medicago sativa* Gene Index 1.2 (MSGI 1.2) is composed of 112,626 unique sequences ranging from 100 bp to 15,768 bp

The MSGI 1.2 transcriptome assembly consists of 112,626 unique sequences (Additional file 1). On average, 15.25 million 76 bp reads were generated for each cDNA library (Additional file 2) and 84 % of the reads generated mapped to the MSGI 1.2 assembly. The average MSGI 1.2 contig size is 1,352 bp, with the largest contig spanning 15,768 bp (Fig. 2). The assembly provides 152,325,272 bp of alfalfa sequence, a significant increase in the publicly available sequence data for this crop. The MSGI 1.2 transcriptome represents 19 % of the predicted 800 Mbp *M. sativa* genome, which is double the percentage of the *M. truncatula* genome predicted to be transcriptionally active (8.3 %) [33], but less than that of Arabidopsis (24 %) [34].

Sequencing transcripts from multiple tissues and subspecies netted a greater overall coverage of the *M. sativa* transcriptome (relative to *M. truncatula*), than either single tissue analyses from multiple alfalfa cultivars (*M. sativa* ssp. *sativa*, *M. sativa* ssp. *caerulea*, and *M. sativa* ssp. *falcata* [29]) or from multiple tissue samples from a single alfalfa cultivar [32]. Sequencing of stems from 27 different alfalfa genotypes by Li et al. [29], resulted in the assembly of 25,183 contigs. Sequencing from 15 different tissues by Liu et al. [32], resulted in the assembly of 40,433 contigs. Sequencing of roots and shoots from *M. truncatula* ssp *sativa* var. Chilean and *M. truncatula* ssp *falcata* var. Wisfal resulted in the assembly of 54,216 sequences, though 454 technology precluded expression analysis [32]. Future transcript profiling experiments in alfalfa focusing on plant samples from multiple genotypes, stages of development, and under biotic and abiotic stress conditions may provide a more thorough alfalfa transcriptome.

### Annotations and functional classifications

Putative annotations for MSGI 1.2 sequences were assigned using BLASTX searches [35] against predicted proteins from seven Phytozome (www.phytozome.net) angiosperm clade anchor species and the UniProt knowledgebase [36] (released April 2014) (for details see Methods). This approach assigned tentative annotations to 90,388 transcripts (80 % of the MSGI 1.2 assembly) (Additional file 3). Of the Phytozome angiosperm clade anchor species, *M. truncatula* proved most similar to the MSGI 1.2 sequences, providing annotations for 79,291 sequences in the *de novo* assembly. The 79,291 MSGI 1.2 sequences annotated by *M. truncatula* corresponded to 70 % of the MSGI 1.2 assembly. *M. truncatula* genes and homologous MSGI 1.2 sequences were visualized on the *M. truncatula* chromosomes (plotted as the number of features within a 150 kb window) to ensure the MSGI 1.2 sequences were evenly distributed across the *M. truncatula* genome (Additional file 4). This visualization identified a number of regions on the *M. truncatula* genome with a significant increase in MSGI 1.2 sequences (ie: the end of Chromosome 6), the majority of which are involved in defense responses. Restricting the BLAST analysis to Arabidopsis for gene ontology (GO) associations identified 64,631 MSGI 1.2 sequences (57 %) with an Arabidopsis homolog.

Alfalfa transcripts corresponding to all protein encoding genes in the two *M. sativa* subspecies were compared to the predicted transcriptomes (primary transcripts) of the related legumes *M. truncatula,* soybean *(G. max)* and common bean (*P. vulgaris).* Soybean has undergone a whole genome duplication event not shared by common bean [37]. Similarly, the transcriptome of alfalfa (a tetraploid) is expected to be twice that of *M. truncatula* (a diploid). BLASTX [35] with an E-value cutoff of 1E-10 was used to compare the primary transcripts of soybean, *P. vulgaris, M. truncatula* and the MSGI 1.2 assembly to predicted proteins in the Arabidopsis genome (TAIR v. 10; www.arabidopsis.org). When we compared *P. vulgaris* and soybean to Arabidopsis, 25,119 of *P. vulgaris* transcripts and 49,304 of soybean transcripts had a hit to a predicted Arabidopsis proteins, clearly reflecting the whole genome duplication event in soybean (Table 1). Similarly, when we compared the MSGI 1.2 assembly and *M. truncatula* transcripts to Arabidopsis, 64,631 MSGI 1.2 transcripts and 35,644 *M. truncatula* transcripts had a hit to a predicted Arabidopsis protein. Given that 85 % of transcripts in MSGI 1.2 are expressed in both B47 and F56, and a similar number of unique Arabidopsis genes were identified for each of the legume species, our data confirms the MSGI 1.2 assembly is complete and the transcriptome of *M. sativa* is twice the size of *M. truncatula*.

**Table 1:** BLAST analyses to Arabidopsis confirm a genome duplication event in *M. sativa*

	*P. vulgaris*	*G. max*	*M. truncatula*	*M. sativa*
Number of primary transcripts	27,197	56,044	50,894	112,626
Transcripts with BLASTX hit to Arabidopsis	25,119	49,304	35,644	64,631
Unique Arabidopsis sequences	14,283	15,407	15,170	15,607

The primary transcripts of *Phaseolus vulgaris*, *Glycine max*, *Medicago truncatula*, and *Medicago sativa* (MSGI 1.2) were compared to the 27,416 predicted primary proteins of Arabidopsis using BLASTX with an E-value cutoff of 1E-10. The ratio of transcripts from each legume species that match an Arabidopsis protein clearly reflects a whole genome duplication event in *G. max* relative to *P. vulgaris* and a polyploidy event in *M. sativa* relative to *M. truncatula*. A similar number of unique Arabidopsis sequences were identified from the BLAST report for each legume, confirming the breadth of the *M. sativa* assembly

**Fig. 3:**
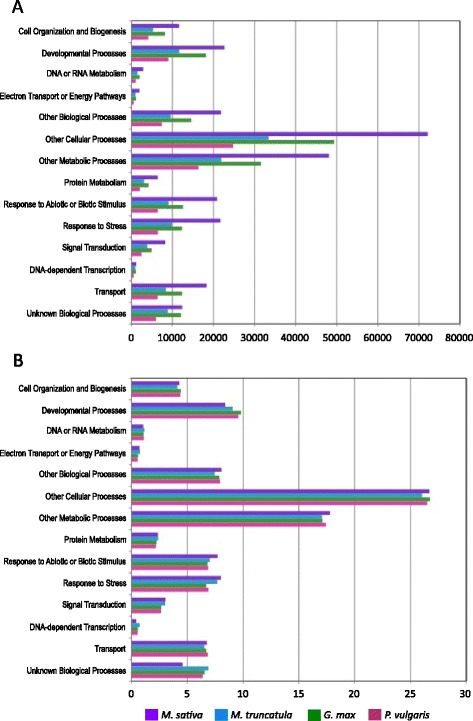
Gene ontology analyses of legume transcripts confirm the breadth of the *M. sativa* transcript assembly. **a** Total number of transcripts in MSGI 1.2 *M. truncatula, G. max,* and *P. vulgaris* with Biological Process (BP) Gene Ontology (GO) Slim annotations*.* The 2-fold difference in transcript number reflects a polyploid event in *M. sativa* compared to *M. truncatula* and a genome duplication event in *G. max* compared to *P. vulgaris*. **b** The percentage of transcripts with each BP GO Slim annotation, from the total number of sequences with BP GO Slim annotations, is consistent across all four legumes, suggesting the MSGI 1.2 assembly represents the majority of transcripts in the *M. sativa* genome

To evaluate the breadth of gene function across the MSGI 1.2 assembly, we compared the gene ontology (GO) [38] slim annotations of the MSGI 1.2 assembly and the primary transcripts of *M. truncatula,* soybean, and *P. vulgaris.* The total number of transcripts associated with each biological process (BP) GO Slim term is approximately 2-fold greater for *M. sativa* and *G. max* than for *M. truncatul*a and *P. vulgaris* (Fig. 3a). These results provide additional evidence of genome duplication event in soybean and a polyploid event in *M. sativa*. The percentage of transcripts within each BP GO Slim annotation is similar in all four legumes (Fig. 3b) confirming the MSGI 1.2 assembly encompasses the majority of transcripts in the *M. sativa* genome.

### Highly expressed and consistently expressed transcripts

Using the expression profiles of both B47 and F56, we identified the 500 most highly expressed transcripts in each subspecies (Additional files 5 and 6). Comparing the top 500 expressed transcripts from each subspecies revealed 336 transcripts were common to both lists including five that were flower-specific and 48 that were nodule-specific. While none of the 48 nodule-specific sequences were annotated, additional analyses confirmed that 29 of the transcripts contain a conserved cysteine cluster motif common to late nodulin genes and nodule cysteine-rich peptides [39]. Because these 48 nodule-specific transcripts are among the most highly expressed transcripts identified in any of the tissue samples and they are also conserved between the two subspecies, they are excellent candidates for further characterization in nodule formation and the establishment of symbiosis in alfalfa.

Transcripts consistently expressed in all tissues may serve ‘housekeeping’ functions. The identification of consistently expressed transcripts is essential for any future qRT-PCR experiment. To aide in identifying potential housekeeping transcripts in MSGI 1.2, sequences with an RPKM >2 in all samples were identified and the co-variance (standard deviation/mean) was calculated. This method identified 50 transcripts with a co-variance <0.13 among all tissues analyzed (Additional file 7). The transcript with the lowest co-variance (COV = 0.098) was contig_8048 with an average RPKM = 8 across all tissues in both *M. sativa* subspecies; however, this transcript was not homologous to any sequence used in the annotation scheme. Contig_67454, which is annotated, has the second most conserved expression pattern, with an average RPKM = 13 across all tissues in both subspecies. This transcript is highly homologous to the *M. truncatula* gene Medtr1g86310.1, a member of the ubiquitin superfamily, suggesting a role in protein degradation. Additionally*,* we identified the 50 transcripts with the most conserved expression patterns in each subspecies (Additional files 8 and 9). In *M. sativa* ssp. *sativa* the most consistently expressed transcript (RPKM = 2 in all tissues) is contig_33355, which is highly homologous to Medtr2g092930.1, and encodes a phosphoenolpyruvate carboxylase 3 subunit. In *M. sativa* ssp. *falcata* the most consistently expressed transcript (RPKM = 2 in all tissues) is contig_65397, which is highly homologous to Medtr4g092900.1, and encodes a PLAC8 protein. These transcripts, raw and normalized expression data, may be useful in identifying stably expressed transcripts in tissues of interest for future quantitative real-time PCR experiments to normalize expression across various alfalfa plant tissues, although consistent expression will need to be confirmed empirically.

### Single nucleotide polymorphism identification

Single nucleotide polymorphisms (SNPs) between MSGI 1.2 and the two *M. sativa* subspecies were identified from RNAseq read alignments to the MSGI 1.2 assembly (for details see Methods). These analyses identified a total of 110,241 SNPs in the MSGI 1.2 assembly (Additional file 10). These SNPs were identified in 17,317 unique MSGI 1.2 sequences (15 % of the MSGI 1.2 assembly). Of the 110,241 SNPs, 38,645 are unique to transcripts derived from B47 and are distributed among 10,861 MSGI 1.2 transcripts while 44,800 are unique to transcripts derived from F56 and are distributed among 11,585 MSGI 1.2 transcripts (Table 2). Of the sequences containing subspecies-specific SNPs, 1,350 transcripts in B47 and 1,389 transcripts in F56 exhibit tissue-specific expression. In addition to subspecies-specific SNPs, there were also 26,796 SNPs from 6,749 MSGI 1.2 sequences that were identified in transcripts from both B47 and F56 (Table 2 and Additional file 10). Since these SNPs were found in both subspecies, it means these contigs likely contain sequences from multiple alleles or closely related genes. Previous studies also identified high frequencies of SNPs in alfalfa transcripts from different genotypes, with a high percentage of SNPs validated to be polymorphic [7, 28, 29]. SNP densities, among MSGI 1.2 sequences corresponding to *M. truncatula* genes, were plotted along the *M. truncatula* chromosomes (Additional file 4). Largely mirroring the gene density plots, SNPs are distributed across all chromosomes. Overall, there is usually an equal number of SNPs from both sub-species and SNPs shared by both sub-species, though there are a few instances where SNPs from one sub-species dominates. MSGI 1.2 sequences corresponding to these instances are candidates for further study to determine if gene families have been collapsed in the MSGI 1.2 assembly, if there is excessive alternative splicing among these sequences, or if these sequences have diverged between the two sub-species. This publicly available SNP data should prove a valuable resource for future marker-assisted selection and genome-wide association studies to identify the genetic underpinnings of complex traits in alfalfa.

**Table 2:** Single nucleotide polymorphism statistics

	SNPs unique to a single subspecies		SNPs in both subspecies
	B47	F56	B47 and F56
Total SNP count	38,645	44,800	26,796
Tissue specific SNPs	23,879	25,875	4,083
MSGI 1.2 sequences with SNPs	10,861	11,585	6,749

Single nucleotide polymorphisms (SNPs) unique to each *M. sativa* subspecies and SNPs identified in both *M. sativa* subspecies (likely representing allelic variation). Roughly 60 % of SNPs unique to one subspecies are only identified in a single tissue. The number of MSGI 1.2 sequences containing SNPs reveals that each sequence contains multiple SNPs. Details on each SNP can be found in Additional file 10

### Web-accessible alfalfa gene index and expression database

We utilized the Alfalfa Gene Index and Expression Database (AGED), available at http://plantgrn.noble.org/AGED/ to assess specific biological processes in flowers, stems, leaves, roots, and nodules. This database was built using a similar architecture as the LegumeIP platform to retrieve and visualize expression patterns using RNA-seq data [40]. To provide the most functionality for alfalfa researchers, AGED allows the user to: (i) search for differentially expressed sequences between the two subspecies and between tissues within a subspecies; (ii) query for transcripts expressed above a user-provided level for each tissue; (iii) obtain graphical and tabular expression profiles across all tissues for both *M. sativa* subspecies for transcripts of interest; and (iv) retrieve the annotation for a transcript of interest. Additionally, all transcript sequence data are available for download and use including: (i) the MSGI 1.2 sequences in FASTA format; (ii) the expression profiles and annotations of MSGI 1.2 sequences from all libraries (each tissue sample from the two subspecies); and (iii) subspecies-specific and subspecies-independent SNPs. This database is fully expandable and new RNA-seq data can be integrated into the database and analysis tools can be updated to provide additional functionality to compare between experiments. This accessibility should facilitate additional mining of the data and a better understanding of biological processes in alfalfa.

### Differential expression between and within subspecies

The phenotypic differences between *M. sativa* ssp*. sativa* (B47) and *M. sativa* ssp*. falcata* (F56) suggest fundamental differences in genomic structure, gene content, and/or gene expression between these two subspecies. In total, 26,053 transcripts (23 % of all transcripts) were differentially expressed between *M. sativa* ssp. *sativa* and *M. sativa* ssp. *falcata* (B47 and F56) (Table 3). In B47, 58,932 transcripts (52 %) were identified as differentially expressed between tissues (Table 4). In F56, 69,143 transcripts (61 %) were differential expressed between the sampled tissues (Table 5).

**Table 3:** Transcripts differentially expressed between *M. sativa* subspecies

Leaf	Flower	ES	PES	Root	Nodule
5,596	6,892	5,636	1,723	2,094	4,112

Transcripts differentially expressed (fold change ≥ 2, FDR ≤ 0.05) between *Medicago sativa* ssp. *sativa* (B47) and *Medicago sativa* ssp. *falcata* (F56) as identified by NOIseq

*ES* elongating stem internodes, *PES* post-elongation stem internodes

**Table 4:** Transcripts differentially expressed between tissue samples in *M. sativa* ssp. *sativa* (B47)

	B47 Leaf	B47 Flower	B47 ES	B47 PES	B47 Root	B47 Nodule
B47 Leaf	-	6,312	7,893	3,612	7,213	6,831
B47 Flower	6,240	-	7,526	3,121	6,989	7,038
B47 ES	5,875	6,321	-	1,083	4,669	6,027
B47 PES	2,174	3,751	8,72	-	3,373	4,857
B47 Root	6,731	7,207	4,846	4,159	-	4,192
B47 Nodule	9,407	1,1284	1,0092	7,186	4,866	-

Transcripts differentially expressed (fold change ≥ 2, FDR ≤ 0.05) between different tissues of *Medicago sativa* ssp. *sativa* (B47) as identified by NOIseq. The number of transcripts in each cell represents transcripts up-regulated in the column tissue compared to the row tissue

*ES* elongating stem internodes, *PES* post-elongation stem internodes

**Table 5:** Transcripts differentially expressed between tissue samples in *M. sativa* ssp. *falcata* (F56)

	F56 Leaf	F56 Flower	F56 ES	F56 PES	F56 Root	F56 Nodule
F56 Leaf	-	8,826	1,1442	7,681	1,1080	9,077
F56 Flower	8,438	-	8,650	5,887	6,486	7,701
F56 ES	9,912	8,337	-	2,447	6,702	16,932
F56 PES	6,123	5,744	1,928	-	5,138	6,131
F56 Root	9,282	8,561	6,148	5,611	-	5,123
F56 Nodule	16,805	18,185	7,360	13,246	11,167	-

Transcripts differentially expressed (fold change ≥ 2, FDR ≤ 0.05) between different tissues of *Medicago sativa* ssp. *falcata* (F56) as identified by NOIseq. The number of transcripts in each cell represents transcripts up-regulated in the column tissue compared to the row tissue

*ES* elongating stem internodes, *PES* post-elongation stem internodes

Subspecies-specific sequences were also identified; including 7,826 transcripts uniquely expressed in at least one tissue sample of B47 but not detected (based on RPKM ≥1) in F56 (Additional file 11) and 8,573 transcripts unique to F56 (Additional file 12). Strikingly, many of the subspecies-specific transcripts identified from each *M. sativa* genotype had tissue-specific expression. Of the 7,826 B47-specific transcripts, 2,944 (37 %) are expressed only in a single tissue while the remaining transcripts are expressed in multiple tissues. Similarly, 2,253 (26 %) F56-specific transcripts exhibit tissue-specific expression patterns while the remaining transcripts are expressed in multiple tissues. GO analyses revealed a statistically significant (*P* ≤ 0.05) over-representation of sequences involved in regulating DNA replication and cell growth and division in sequences unique to B47 (including GO:000678, RNA-dependent DNA replication; GO:0022619, cell differentiation; GO: 0000082, G1/S transition of mitotic cell; GO:0001558, regulation of cell growth; and GO: 0006261, DNA dependent DNA replication) (Additional file 13). Conversely, sequences involved in defense responses (including GO:0006952, defense response; GO:0072953, reactive oxygen species metabolic process; and GO:0009626, plant-type hypersensitive response), phosphatidylinositol signaling (GO:0048015, phosphatidylinositol-mediated signaling; GO:0046855, inositol phosphate dephosphorylation; GO:0032957, inositol triphosphate metabolic processes; and GO:0046854, phosphatidylinositol phosphorylation), and arabinose metabolism (GO:0046373) are significantly (*P* ≤ 0.05) over-represented among sequences unique to F56 (Additional file 14). Many of the GO categories over-represented in F56-specific sequences are involved in inositol homeostasis. In plants, increased phosphatidylinositol results in increased starch content and impacts both carbon metabolism and responses to environmental stress [41, 42]. Transcripts involved in both cell shape and cell plate formation are also over-represented among F56-specific transcripts (GO:0008360 and GO:0000911, respectively). Hydroxyproline-rich glycoproteins and arabinogalactan proteins are involved in cell-to-cell interactions, cell proliferation, cell expansion, and cell wall strengthening. Arabinose is an important constituent of hydroxyproline-rich glycoproteins [43]. In Arabidopsis, knocking out arabinose biosynthetic genes results in altered cell shape and cell plate formation [43]. These subspecies-specific transcripts may represent genes important in conferring the contrasting shoot architectures of *M. sativa* ssp. *sativa* and *M. sativa* ssp. *falcata* (Fig. 1). Details of the differentially expressed transcripts can be identified using the AGED website available at http://plantgrn.noble.org/AGED/.

### Cold tolerance

*Medicago sativa* ssp. *falcata* originated in central Asia, a much colder region than the Near East where *Medicago sativa* ssp. *sativa* originated [5]. These ancient geographic origins have imbued the subspecies with distinct cold tolerance profiles. In general, *M. sativa* ssp. *falcata* has greater cold and freezing tolerance than *M. sativa* ssp *sativa*. Previous studies, performed on leaf and crown tissues [44, 45], have identified a number of candidate genes conferring cold tolerance based on their increased expression profiles in *M. sativa* ssp. *falcata* compared to *M. truncatula* under cold-stress conditions. Although the samples in our study were not cold-treated, we identified transcripts in the MSGI 1.2 assembly corresponding to previously identified sequences and examined their expression patterns between B47 and F56 in the six tissues evaluated in the study. As expected, the majority of the sequences had similar expression patterns in the tissues of both subspecies. However, 38 sequences previously identified as cold-induced in *M. sativa* ssp. *falcata* were differentially expressed between B47 and F56 under greenhouse growth conditions. The majority (32) of these sequences were more highly expressed in multiple tissues of *M. sativa* ssp. *falcata* (F56), the more cold-tolerant plant (Additional file 15). Two of these sequences were not assigned annotations. The 30 annotated sequences encode DREB transcription factors (9 sequences), proline dehydrogenases (2 sequences), sucrose synthases (4 sequences), and sequences involved in either gibberellin or glutamate biosynthesis (6 and 7 sequences, respectively). These sequences may be expressed constitutively higher in *M. sativa* ssp. *falcata* compared to *M. sativa* ssp. *sativa*, perhaps in anticipation of cold stress. Only six of the sequences were expressed higher in B47 and all six were differentially expressed between the leaves of the two subspecies. The two annotated sequences both encode sucrose synthase. Four additional sucrose synthase transcripts were differentially expressed between the two subspecies, and in all tissues exhibiting differential expression they were more highly expressed in *M. sativa* ssp. *falcata* (F56). Increased sugar accumulation has been correlated with increased cold tolerance in a number of species [46–51]. Wolkers et al. [46] proposed sugars interact with dehydrin and cold responsive proteins to form stable glasses to prevent desiccation.

### Floral pigmentation

**Fig. 4:**
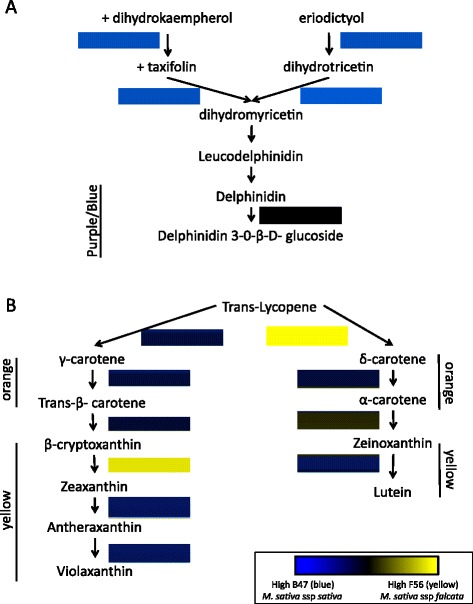
Expression of transcripts conferring flower color in *Medicago sativa* ssp. *sativa* (B47) and *Medicago sativa* ssp. *falcata* (F56). Expression patterns comparing B47 and F56 are presented as heat map blocks. **a** Anthocyanin biosynthesis. Early in the pathway, transcripts are up-regulated 2-fold in B47 compared to F56 (blue blocks). This results in increased delphinidin, the anthocyanin that confers blue coloration to flowers. **b** Carotenoid biosynthesis. Transcripts involved in the conversions of trans-lycopene to δ-carotene and β-cryptoxinthin to zeantin, are up regulated 17- and 6-fold, respectively, in F56 (yellow blocks). The increased carotene synthesis is responsible for the orange and yellow flower color, characteristic of F56

One of the most notable differences between *M. sativa* ssp. *sativa* and *M. sativa* ssp. *falcata* is flower color. *M. sativa* ssp. *sativa* has violet to lavender-colored flowers while *M. sativa* ssp. *falcata* has orange to yellow-colored flowers (Fig. 1). Alfalfa cultivars with mixtures of *M. sativa* ssp. *sativa* and *M. sativa* ssp. *falcata* express a range of flower colors including purple, yellow, cream, white and variegated (ranging from very dark blue to a green or yellow green). Anthocyanins are the primary pigments contributing to violet and blue flowers while orange and yellow flowers are a result of increased carotenoid synthesis. The biochemical pathways of both anthocyanin and carotenoid biosynthesis are well characterized [52–55] but the expression profiles of these sequences in alfalfa have not been previously investigated. Using the MSGI 1.2 assembly and RNA-seq profiles, we examined the expression patterns of transcripts involved in the biosynthesis of floral pigments in both subspecies. In floral tissues of *M. sativa* ssp. *sativa* (B47) the transcript encoding a flavone 3-dioxygenase converting dihydrotricetin to dihydromyricetin (contig_11784) was up-regulated two-fold compared to the floral tissues of F56 (Fig. 4a). Dihydromyricetin is a precursor to delphinidin, the anthocyanin responsible for blue/purple coloration. Conversely, in F56 the transcript for lycopene ε-cyclase, which converts trans-lycopene to δ-carotene and the transcript for ß-cryptoxanthin 3′-hydroxylase, which converts ß-cryptoxanthin to zeaxanthin (both imparting orange or yellow coloration to floral tissues) were 17- and 6-fold higher relative to B47 (Fig. 4b). The data from this study indicate both anthocyanin and carotenoid pathways are expressed in the floral tissues of both subspecies, suggesting that it is the relative expression of these genes and/or enzyme activities that are responsible for the flower colors exhibited by B47 and F56. These results lend genetic support to extend the earlier biochemical work that found evidence for activity of both pathways in yellow and purple flowers of diploid alfalfa [56]. That analysis found that yellow flower color in *M. sativa* ssp. *falcata* was largely due to carontenoid xanthophyll esters and that the quercetin pigments from the anthocyanin synthesis pathway had minor phenotypic effects. In contrast, purple alfalfa flowers contained a mixture of three anthocyanins (delphinidin, petunidin, and malvidin) and color variation was due to background effects of the xanthophyll pigments and their interactions with anthoxanthin pigments, rather than differences in the anthocyanin content [56].

### Stem and cell wall related sequences

Although alfalfa stems contain large amounts of cell-wall carbohydrates (approximately 70 % of stem dry weight), the majority of the cell walls, specifically the cellulose and hemicellulose fractions, are poorly digested by ruminant animals, resulting in inefficient use of the forage [57]. Lignification of cell walls in post-elongation internodes poses a barrier to cell wall polysaccharide digestion. Industrial conversion of stems to ethanol or other fermentation products from cell wall polysaccharides suffers from similar constraints due to lignification. Increasing the digestibility of alfalfa cell walls would improve animal performance and utility of alfalfa as a biomass feedstock. However, progress to improve digestibility has been slow due to the lack of information on stem cell wall related genes and their regulation.

B47 was selected from germplasm developed for use as a biomass energy feedstock with large, erect non-lodging stems [58]. In contrast, F56 was selected from germplasm developed for increased forage yield with recumbent stem architectures. Based on these phenotypic differences, the expression of genes involved in cellulose and lignin synthesis was expected to differ between the two subspecies. Comparing B47 and F56 identified 5,636 and 1,723 differentially expressed transcripts in elongating and post-elongation stem internodes, respectively (Table 3). In elongating internodes 10 cellulose synthase transcripts were differentially expressed between the two subspecies, all of which were expressed at a higher level in B47. Conversely, a single (different) cellulose synthase-encoding transcript was differentially expressed in post-elongating internodes and was expressed at a higher level in F56 than B47.

Using the MSGI 1.2 annotations, we identified 327 sequences involved in lignin biosynthesis (data not shown). Of these, 96 (30 %) were differentially expressed between B47 and F56 in elongating and/or post-elongating stem internodes. In elongating stem internodes, 39 transcripts were up-regulated in B47 and 49 transcripts were up-regulated in F56. In post-elongating stem internodes 33 transcripts were up-regulated in B47, while 44 transcripts were up-regulated in F56. In elongating internodes, three transcripts encoding ferrulate-5-hydroxylase (F5H) have greater expression in F56 while two have greater expression in B47. In post-elongating stems three F5H transcripts are expressed at higher levels in B47 while only a single F5H encoding transcript is expressed higher in F56. F5H is involved in syringyl lignin biosynthesis. In Arabidopsis, F5H over-expression results in increased stem lignin [59]. Two transcripts encoding caffeic acid O-methyltransferase (COMT), which acts downstream of F5H in the lignin biosynthesis pathway, were differentially expressed between B47 and F56. Both were expressed at higher levels in elongating and post-elongating stem internodes of B47. In alfalfa, down-regulating COMT results in decreased lignin content [60]. Further dissection of gene expression differences leading to differences in alfalfa stem cell wall composition will likely require isolation of specific tissue types as demonstrated by Zhao et al. [61].

Using the MSGI 1.2 annotation, 157 NAC transcription factors (TFs) were identified in the transcriptome assembly. The majority (138) were expressed in elongating and/or post-elongating stem internodes, suggesting they are important in alfalfa stem development. Four NAC TFs were uniquely expressed in elongating and post-elongating stem internodes (in both subspecies). All four had higher expression in the post-elongating internodes of both subspecies, although the change in expression was not statistically significant in any comparisons. Genomic studies in Arabidopsis and poplar have identified NAC domain TFs that regulate the formation of xylem and secondary cell wall formation [62, 63]. In this study, five NAC TFs were differentially expressed between B47 and F56 elongating internodes, three up-regulated in F56 and two up-regulated in B47. Two of the NACs differentially expressed between B47 and F56 (both up-regulated in F56) may play important roles in secondary cell wall development. Contig_63859, which is expressed eight times higher in F56 than B47, is homologous to the *A. thaliana* gene At4g28500 (*SND2)*. SND2 is an indirect target of SND1, a master regulator of xylem fiber secondary cell wall formation [64]. In Arabidopisis, *SND1* repression results in decreased cell wall sugars (glucose, mannose, and xylose) while *SND1* over-expression results in a xylem fiber-specific increase in the secondary cell wall thickness accompanied by an increase in the mannose and rhamnose content of stems [64]. Additionally, contig_62770, which was expressed three times higher in F56 than B47, is homologous to the Arabidopsis gene At5g13180, *VNI2*. In Arabidopsis, *VNI2* loss of function results in an increased expression of genes involved in xylem vessel formation [65]. Interestingly, the Arabidopsis homologs of the two NAC TFs up-regulated in B47 (contig_23714 and contig_112115) are involved in defense responses [66, 67], but have not been shown to play a role in secondary cell wall development.

We used a single linkage clustering analysis (see Methods for details) to determine if any gene families in *M. sativa* underwent a familial expansion relative to *M. truncatula,* as has been documented in soybean [68], and whether any of these were involved in cell wall synthesis. Using this approach, we identified a total of 138 gene families that have undergone a statistically significant (*P* ≤ 0.01) expansion (or contraction) in *M. sativa* relative to *M. truncatula* (Additional file 16). Three gene families identified in this analysis are directly involved in secondary cell wall synthesis. In Arabidopsis, *cyt1* mutants are deficient in mannose-1-phosphate guanylyltransferase and exhibit a 5-fold decrease in cellulose content [69]. Analysis of *cyt1* mutant cell walls found decreased mannose and fucose (by 40 %) levels, but increased rhamnose and xylose (152 and 122 %, respectively) compared to wild type [69]. Single linkage cluster analyses identified three *cyt1* genes (mannose-1-phosphate guanylyltransferases) in *M. truncatula* and 19 *cyt1* homologs in MSGI 1.2. Examining the expression patterns in B47 revealed that members of this family were expressed higher in elongating stem internodes while in F56 members were more highly expressed in post-elongating stem internodes. The β-D-xylosidase family (cluster 3258: two members in *M. truncatula*, 16 members in MSGI 1.2) exhibits similar expression patterns (increased expression in elongating stem internodes of both B47 and F56, with expression remaining high in the post-elongating internodes of F56). Xylan is the major component of hemicelluloses, the main constituent of plant cell walls. β-D-xylosidase is a key enzyme for xylan degradation [70]. In Arabidopsis, *XYL1* knockout plants show increased susceptibility to hydrolysis by cellulases, suggesting that XYL1 is important in cell wall structure [70]. Finally, the *WAT1* gene family (cluster 920) exhibited a fourfold expansion in *M. sativa* (two members in *M. truncatula*, 17 in MSGI 1.2). WAT1 acts upstream of SND1 and NST1 to regulate secondary wall formation in xylem fibers [71]. In Arabidopsis, knockout mutants exhibit no secondary cell wall development in xylem fibers, are shorter, bushier and have twice as many stems and stems are weaker [71]. *WAT1* expression is highly up-regulated in both elongating and post-elongating stem internodes of both B47 and F56. The familial expansion of these genes in *M. sativa* subspecies, coupled with their increased expression in both elongating and post-elongating internodes, make these sequences excellent candidates for future experiments investigating alfalfa cell wall development.

### Nodules

Legumes have a unique ability to establish and sustain symbiotic relationships with rhizobia to form N_2_ fixing nodules. This symbiotic relationship requires signaling between the plant and bacteria for nodule development and nodule maintenance. Many of the genes involved in nodulation have been identified in *M. truncatula* [72–75]. Using BLASTN (E-value cutoff of 1E-10), we identified homologs of the nodulation genes in the MSGI 1.2 assembly (Additional file 17). As expected, expression was usually highest in nodules and associated roots. In *M. truncatula*, a strikingly large number (~300) of nodule-specific cysteine-rich peptides (NCRs) with a conserved pattern of cysteine residues have been identified [39, 76], some of which have a demonstrated role in nodule development [77, 78]. NCRs have not been previously investigated in alfalfa. Using all six reading frame translations of the MSGI 1.2 assembly, we identified 1,330 nodule-specific sequences with a nodule-specific NCR motif (Additional file 18). This is almost exactly four times the number identified in *M. truncatula*, further confirming that this gene family did not undergo a familial gene expansion, which may indicate highly conserved function within the NCR family. The 1,330 nodule-specific NCR sequences are roughly 22 % of the nodule-specific sequences identified in both *M. sativa* subspecies. Interestingly, 21 nodule-specific sequences containing NCR motifs were expressed in B47 but were not expressed in F56 while another 30 sequences exhibited the opposite expression pattern. Of these 51 sequences, 20 encoded a signal peptide [79], a hallmark of NCR sequences (Additional file 18). The remaining 31 sequences are most probably NCR sequences, but are not full-length sequences likely due to the RNA extraction processes. These 51 NCRs may be sub-species-specific or may reflect different NCRs expressed at various stages of nodule development.

Of all the tissue-specific sequences (23,725) almost 40 % were nodule-specific (9,360; Additional file 19) (Table 6). Not surprisingly, sequences with annotations involving transport and nitrogen homeostasis, the major function of nodules, are significantly (*P* < 0.05) over-represented among nodule-specific sequences (Additional file 20). The high number of transcripts involved in transport, including amino acid transporters, peptide transporters, iron and sulfur transporters, transmembrane transporters, sugar transporters, and nitrate transporters, reflect the importance of photosynthates imported to the nodule and nitrogenous compounds exported from the nodule to the roots for use throughout the plant. Additionally, 45 transcription factors from 12 different families (AS2, Aux/IAA, bHLH, bZIP, C2H2, CCAAT, G2, Homeobox, MADS box, MYB, NIN, and WRKY) exhibit nodule-specific expression in both B47 and F56. Nodule-specific expression of transcription factors highlights the changes in gene expression required to accommodate nodulation and symbiosis.

**Table 6:** Transcript expression profiles in *Medicago sativa* ssp. *sativa* (B47) and *M. sativa* ssp. *falcata* (F56)

Tissue	*M. sativa* ssp. *sativa* (B47)		*M. sativa* ssp. *falcata* (F56)		Common
	Expressed	Tissue-specific	Expressed	Tissue-specific	Tissue-specific
Leaf	71,003	1,712	74,251	1,233	255
Flower	78,163	3,970	79,320	4,199	2,919
ES	77,220	581	74,612	475	70
PES	73,768	293	76,693	332	52
Root	77,840	3,374	76,167	2,821	1,329
Nodule	75,442	7,491	72,594	7,605	5,736
Total	104,787	17,421	104,040	16,665	All Total: 112,626

Transcripts must have an RPKM ≥1 to be considered expressed. Tissue-specific transcripts have an RPKM ≥1 in a single tissue but an RPKM <0 in all other tissues. The number of tissue-specific transcripts common to both *M. sativa* subspecies is denoted in the common column. Total number of transcripts expressed in each sub-species and transcripts that are tissue specific are denoted in the last row

*ES* elongating stem internodes, *PES* post-elongation stem internodes

Among the 5,736 sequences that exhibit nodule specific expression in both subspecies, sequences involved in cyanoalanine metabolism were significantly (*P* < 0.05) overrepresented in nodules. Cyanoalanine nitrilase (GO:0047427) is important in cyanide detoxification, converting cyanoalanine into aspartic acid [80]. Similarly, cyanoalanine hydratase (GO:0047558), a *NIT4* homolog, hydrolyzes cyanoalanine into asparagine and aspartic acid [81]. In *Neurospora crassa*, NIT4 regulates the expression of nitrate assimilatory structural genes [82]. Cyanide is produced as a by-product of ethylene biosynthesis, which is regulated by the availability of nitrate in the soil. Increased ethylene inhibits nodule and lateral root formation. It is possible that enzymes involved in cyanide catabolism serve as an alternative asparagine synthesis pathway, induced as the plant shifts from N_2_ fixation to utilizing nitrate available in surrounding soils. Asparagine is produced from aspartic acid, the primary assimilation product of symbiotic nitrogen fixation [83] and has recently been implicated in the N-feedback regulation of N fixation in *M. truncatula* [84]. Finally, sequences encoding glutamine synthase (GO:0006541), one of the main enzymes for assimilating symbiotically fixed nitrogen [85], are over-represented among nodule-specific transcripts compared to the rest of the MSGI 1.2 assembly.

Although many sequence families are over-represented in nodule-specific transcripts, there are also many sequences that were not assigned an annotation by any method. However, the conservation of tissue-specific expression between two subspecies is indicative of conserved function. These nodule-specific sequences are excellent candidates for future work exploring nitrogen fixation in *M. sativa*.

## Conclusion

The data presented in this study have been assembled into an online resource for analyses of gene expression in *M. sativa* for six distinct tissue types derived from two biologically distinct subspecies (*M. sativa* ssp. *falcata* and *M. sativa* ssp. *sativa*). This publicly available resource, AGED (available at http://plantgrn.noble.org/AGED/), is a valuable tool for alfalfa and legume researchers investigating various biological processes. To illustrate the utility of this resource, we used the data to explore the differences between the two *M. sativa* subspecies in their expression of sequences involved in cold tolerance, anthocyanin and carotenoid biosynthesis as it relates to flower color, identified sequences involved in shoot architecture, and present nodule-specific sequences which may play important roles in nitrogen fixation.

## Methods

### Plant materials and growth conditions

The *M. sativa* ssp*. sativa* genotype B47 was selected from a population developed for high biomass production [58] and the *M. sativa* ssp. *falcata* genotype F56 was selected from a population developed for increased forage yield [86]. All plants were propagated and grown in a greenhouse. Stem cuttings were rooted in vermiculite for 14 days then transferred to six inch pots with a pasteurized soil:sand (2:1) mixture. Eight clones of B47 and F56 were propagated for each of the three biological replicates. Plants were inoculated with *Sinorhizobium meliloti* strain 102 F51 and watered daily. Once a week plants were watered with 0.25X Hoagland’s nutrient solution [87] containing 50 ppm N. Plants were allowed to flower, then cutback twice. Flowers, leaves, elongating stem internodes, and post-elongation internodes were harvested 28 days after the second cutback as described previously [22]. Both sub-species were flowering at the time of harvest. Root and nodule samples were obtained from cuttings transplanted into ten-inch pots containing quartz sand. For the first 3 days after transfer, the pots were watered with 0.5X Hoagland’s nutrient solution containing 100 ppm N. On day four, the pots were flushed with water to remove all nutrients and inoculated with *S. meliloti* strain 102 F51. From day five until harvest (23 days after inoculation) the pots were watered daily with 0.5X Hoagland’s nutrient solution without nitrogen. Nodules and apical root tips were harvested 23 days after inoculation. All samples were immediately frozen in liquid nitrogen and stored at −80 °C until used for RNA extraction.

### RNA extraction, cDNA library preparation, and sequencing

Total RNA was extracted from individual tissue samples (leaves, roots, nodules, flowers, elongating internodes, and post-elongation internodes) using the Qiagen RNeasy Kit (Qiagen, Valencia, CA). cDNA library preparation and sequencing reactions were performed at the University of Minnesota Genomics Center. Illumina library preparation, clustering, and sequencing reagents were used throughout the process following the manufacturer’s recommendations. Briefly, mRNAs were purified using poly-T oligonucleotide-attached magnetic beads and then fragmented. The first- and second-strand cDNAs were synthesized and end-repaired. Adaptors were ligated after adenylation at the 3′ ends. After gel purification, cDNA templates were enriched by PCR. cDNA libraries were validated using a High Sensitivity Chip on the Agilent 2100 Bioanalyzer (Agilent Technologies, Santa Clara, CA). The cDNA libraries were clustered on flowcells using an Illumina automated clonal cluster generator (cBOT). After clustering, samples were loaded on the Illumina GA-II machine. A single lane was used for each cDNA library for the first replicate. Libraries for the second and third replicates were barcoded and two libraries were run on a single lane. Samples were sequenced as single end reads with 76 cycles. Initial base calling and quality filtering of the Illumina GA-IIx image data were performed using the default parameters of the Illumina GA Pipeline GERALD stage (Illumina, San Diego, CA). A total of 546,157,051 single-end 76-bp reads were generated from the 36 samples. After filtering homopolymers and short reads (less than 76 bp), 461,582,298 reads were retained for further analysis. Read quality was evaluated and reads were trimmed to a minimum quality score of 35 using a custom Perl script. After trimming, reads less than 15 bp were discarded. Reads are available at the NCBI short read archive database as accession SRP055547.

### *de novo* transcriptome assembly and annotation

A *de novo* assembly was produced by combining reads retained after quality control analysis using Velvet/Oases [88, 89] version 1.2.03 and a k-mer of 27. Velvet was allowed to predict the expected coverage level and optimal coverage cutoff. A coverage cutoff of 12, edge fraction cutoff equal to 0.75, degree cutoff of two, and minimum transcript length of 200 bp were applied using Oases. To reduce sequence redundancy, sequences were collapsed using CAP3 and cdHIT [90–92]. Previous studies associated low read count with false positive expression profiles [93, 94]. To mitigate this, sequences were required to have at least 10 reads mapping to the sequence in two of the three biological replicates to be retained. This yielded a final sequence assembly of 112,626 sequences.

Putative functions were assigned to the MSGI 1.2 sequences by conducting BLASTX queries against the predicted protein sequences of anchor clades of Phytozome (www.phytozome.net) including Arabidopsis (*A. thaliana*, v. 10, [34]), Medicago (*M. truncatula,* version 4.0v1, [33]), soybean (*G. max,* v. 2.0 [68]), eucalyptus (*E. grandis*, v.1.1, [95]), goldsmith (*Aquilegia coerulea* v. 1.1), cassava (*Manihot esculenta* v. 4.1 [96]), and potato (*Solanum tuberosum* v. 3.4 [97]) using an E-value cut-off of 1E-10. The top BLASTX hit for each species was assigned to each of the MSGI 1.2 sequences. MSGI 1.2 sequences were also used in a BLASTX comparison to Arabidopsis to assign descriptive annotations and gene ontology (GO) terms for GO analysis. Additionally, the MSGI 1.2 sequences were queried against the UniProt Knowledgebase (released April, 2014) [36] using BLASTX and an E-value cut-off 1E-10. Overall, this methodology assigned putative annotations to 80 % of the MSGI 1.2 sequences. Annotations of transcripts discussed throughout the manuscript were manually curated to ensure accuracy.

To understand gene ontology biological processes [38] associated with different samples in our dataset we used a Fisher’s exact test [98] with a Bonferroni [99] correction to compare gene ontology within a specific dataset relative to the entire MSGI 1.2 assembly. Note that single genes could be associated with multiple over-represented GO terms.

To allow comparisons between the MSGI 1.2 assembly and the genomes of *M. truncatula* (v.4.0v1), *G. max* (v2.0), and *P. vulgaris* (v.1.0), we compared all assemblies to the Arabidopsis genome (v.10). For MSGI 1.2, BLASTX [35] (E-value cutoff of 1E-10) was used to identify the best Arabidopsis homolog. For *M. truncatula*, *G. max,* and *P. vulgaris*, annotation information was downloaded from Phytozome10 (http://phytozome.jgi.doe.gov/pz/portal.html) and was used to identify Arabidopsis homologs. Custom perl scripts were then used to assign GO information based on the best Arabidopsis homolog (www.arabidopsis.org, version 02/27/14).

We developed GO and KEGG treeviews to facilitate systematic and genome-scale exploration of assembled transcripts. Initial transcript annotations were enhanced using the GSEAServer: a web tool for annotation and enrichment analysis of *de novo* assembled transcripts from non-model plants (http://plantgrn.noble.org/GSEAserver). Default parameters were used to associate MSGI 1.2 GO and KEGG annotations with reference sequence databases. Treeviews are accessible at the AGED website, http://plantgrn.noble.org/AGED/.

### Expression analysis

The expression of each transcript in each of the 36 libraries was determined by calculating the number of 76-bp Illumina reads that mapped to each of the MSGI 1.2 sequences using the Bowtie2 program with default parameters [100]. Raw expression counts were normalized using the RPKM method [101, 102] with custom R scripts as described previously [12]. Multiple studies in a variety of species, including alfalfa [14, 24, 28, 102–105], have illustrated the consistent correlation between gene expression measured by RNA-seq and by qRT-PCR, rendering this validation redundant. Transcripts exhibiting differential expression between samples were identified using the NOIseq program in R [106]. Differentially expressed transcripts were identified using a fold change ≥2 and a *P*-value, corresponding to a false discovery rate (FDR), ≤0.05. Sequence data and lists of differentially expressed transcripts can be accessed at AGED (http://plantgrn.noble.org/AGED/).

To identify gene family expansions within MSGI 1.2 relative to *M. truncatula,* we used single linkage clustering [39]. Transcripts from MSGI 1.2 and *M. truncatula* (primary transcripts only) were combined in a single FASTA file. Sequences from each species were flagged to allow easy identification. BLASTN [35] (E-value cutoff of 1E-20) was used to compare the combined file against itself. Custom perl scripts were used to identify putative gene families at an E-value cutoff of 0. Any sequences with overlapping blast hits were assigned to the same gene family. A Chi-square test with a Bonferroni correction [99] was used to identify gene families with statistically significant expansion in *M. sativa,* taking into account the *M. sativa* polyploid event relative to *M. truncatula.*

### SNP identification

SNPs were identified using mpileup function of SAMTools. Briefly, reads from each sample were mapped to the MSGI 1.2 transcriptome. SNPs represented by at least 10 reads were identified for each biological replicate. To reduce false positives, SNPs were required to be in all three biological replicates to be retained. SNPs identified in a tissue of one subspecies, but not the other, were considered genotype dependent (110,721). An additional 20,833 SNPs were identified in the same tissue of both subspecies, likely representing allelic variation within the transcripts.

### Availability of supporting data

The data supporting the results of this article are available in the NCBI short read archive database (www.ncbi.nlm.nih.gov/sra/) as accession SRP055547. In addition, both the MSGI 1.2 sequences and annotations, including expression data, can be downloaded at http://plantgrn.noble.org/AGED/Download.jsp. Other supporting data are included as additional files.

## Additional files

Additional file 1:
**Sequences of transcripts (fasta seqs) comprising MSGI 1.2 assembly.**
Additional file 2:
**Summary statistics of transcript assembly and mapping from each library.**
Additional file 3:
**MSGI 1.2 transcript expression and annotation.**
Additional file 4:
**MSGI 1.2 features aligned to the** ***M. truncatula*** **(version 4.0) chromosomes.**
Additional file 5:
**Expression and annotation of the 500 most highly expressed transcripts in** ***M. sativa*** **ssp.** ***sativa*** **(B47).**
Additional file 6:
**Expression and annotation of the 500 most highly expressed transcripts in** ***M. sativa*** **ssp.** ***falcata*** **(F56).**
Additional file 7:
**Housekeeping transcripts for MSGI 1.2.**
Additional file 8:
**Housekeeping transcripts for** ***M. sativa*** **ssp.** ***sativa*** **(B47).**
Additional file 9:
**Housekeeping transcripts for** ***M. sativa*** **ssp.** ***falcata*** **(F56).**
Additional file 10:
**SNP data includes contig ID, position, reference and alternate sequence, tissue with SNP,** ***M. sativa*** **subspecies with SNP, transcript expression profile and annotation.**
Additional file 11:
***M. sativa*** **ssp.** ***sativa*** **(B47) specific transcripts.**
Additional file 12:
***M. sativa*** **ssp.** ***falcata*** **(F56) specific transcripts.**
Additional file 13:
***M. sativa*** **ssp.** ***sativa*** **(B47) specific GO statistics.**
Additional file 14:
***M. sativa*** **ssp.** ***falcata*** **(F56) specific GO statistics.**
Additional file 15:
**Expression profile of sequences identified as cold-inducible in** ***M. sativa*** **ssp.** ***falcata*** **in previous studies.**
Additional file 16:
**Gene families with statistically significant expansion in the MSGI 1.2 assembly compared to** ***M. truncatula.***
Additional file 17:
**Expression profiles of sequences involved in nodulation.**
Additional file 18:
**Nodule-specific sequences with a cysteine cluster motif.**
Additional file 19:
**Transcripts exhibiting tissue-specific expression patterns.**
Additional file 20:
**GO statistics for nodule-specific transcripts.**
