# Supplementary figures and images for: The Medicago sativa gene index 1.2: a web-accessible gene expression atlas for investigating expression differences between Medicago sativa subspecies
Source: BMC Genomics. 2015 Jul 7;16(1):502. doi: 10.1186/s12864-015-1718-7 (PMC4492073; doi:10.1186/s12864-015-1718-7)

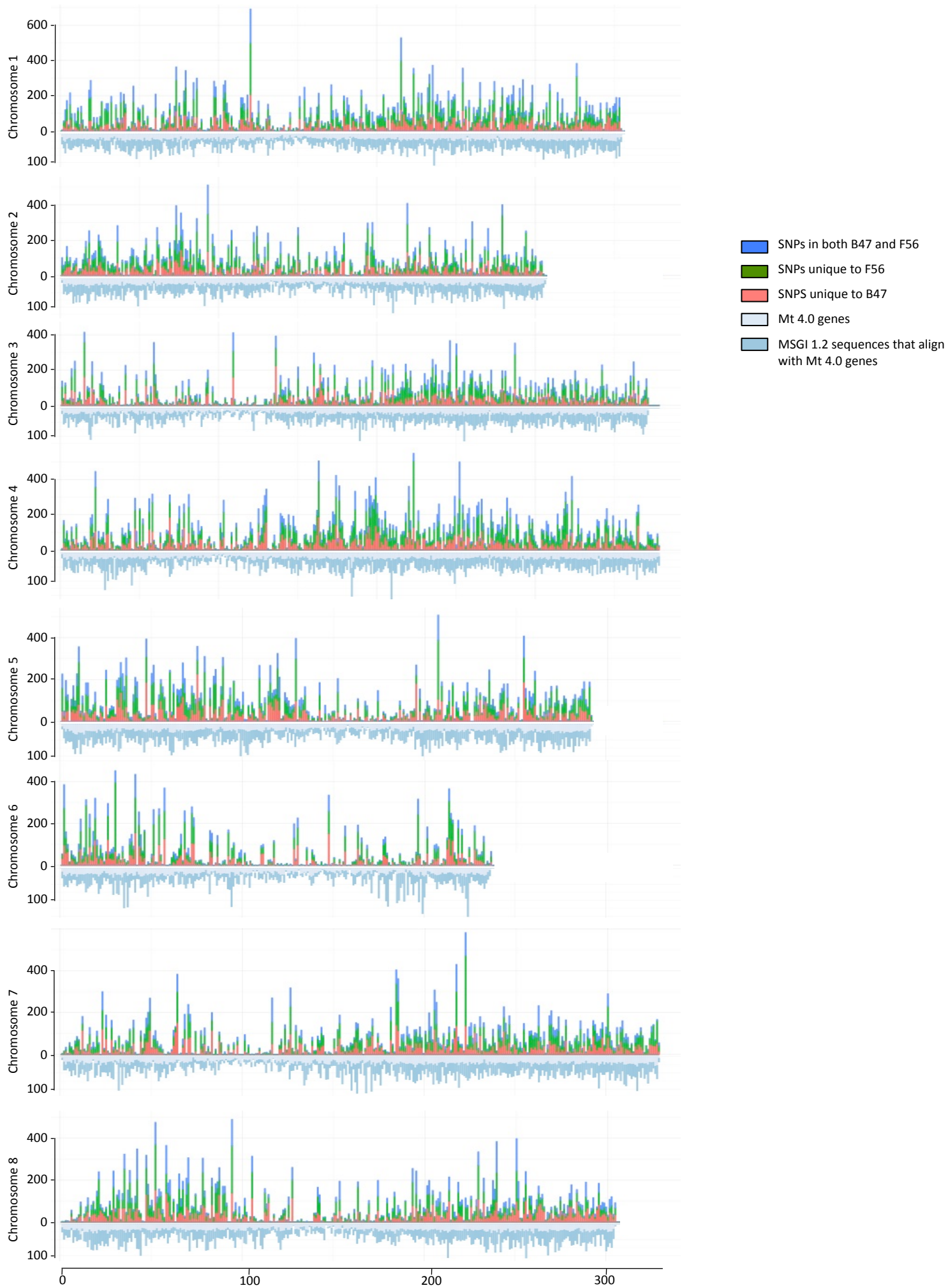

Supplement: Additional file 4: — MSGI 1.2 features aligned to the M. truncatula (version 4.0) chromosomes. [file 12864_2015_1718_MOESM4_ESM.pdf]
