# Supplementary material for: The Medicago sativa gene index 1.2: a web-accessible gene expression atlas for investigating expression differences between Medicago sativa subspecies
Source: BMC Genomics. 2015 Jul 7;16(1):502. doi: 10.1186/s12864-015-1718-7 (PMC4492073; doi:10.1186/s12864-015-1718-7)
Supplement: Additional file 15: — Expression profile of sequences identified as cold-inducible in M. sativa ssp. falcata in previous studies. [file 12864_2015_1718_MOESM15_ESM.pdf]

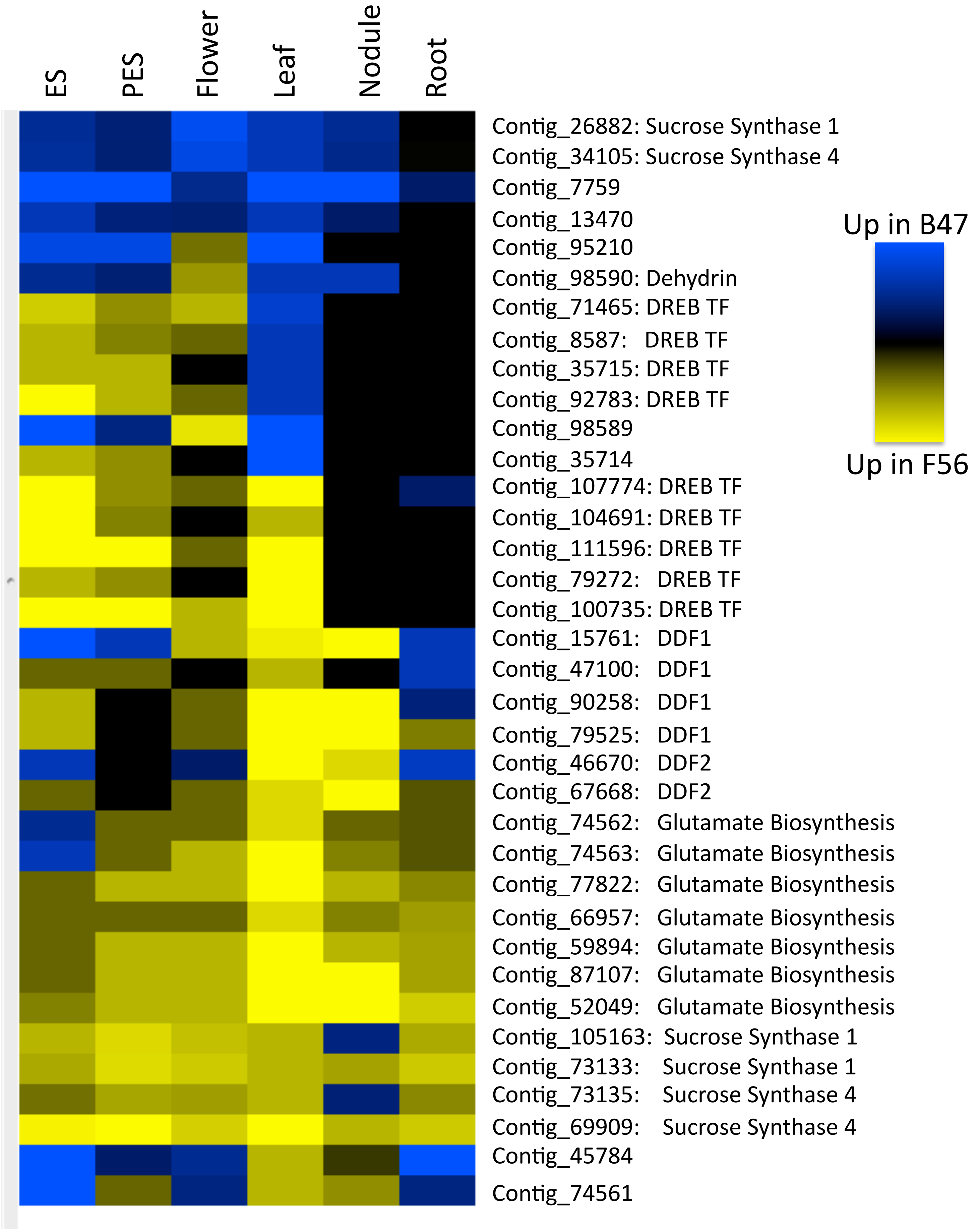

Additional file 15. Expression profile of sequences previously identified as cold inducible in *M. sativa* ssp. *falcata* [33,34], but which are ≥ 2-fold different between B47 and F56 under normal growth conditions. Expression is depicted as fold change of F56/B47. DDF1 (dehydration response element), DREB (DRE binding factor).
