# Supplementary material for: The Medicago sativa gene index 1.2: a web-accessible gene expression atlas for investigating expression differences between Medicago sativa subspecies
Source: BMC Genomics. 2015 Jul 7;16(1):502. doi: 10.1186/s12864-015-1718-7 (PMC4492073; doi:10.1186/s12864-015-1718-7)
Supplement: Additional file 17: — Expression profiles of sequences involved in nodulation. [file 12864_2015_1718_MOESM17_ESM.pdf]

Additional file 17

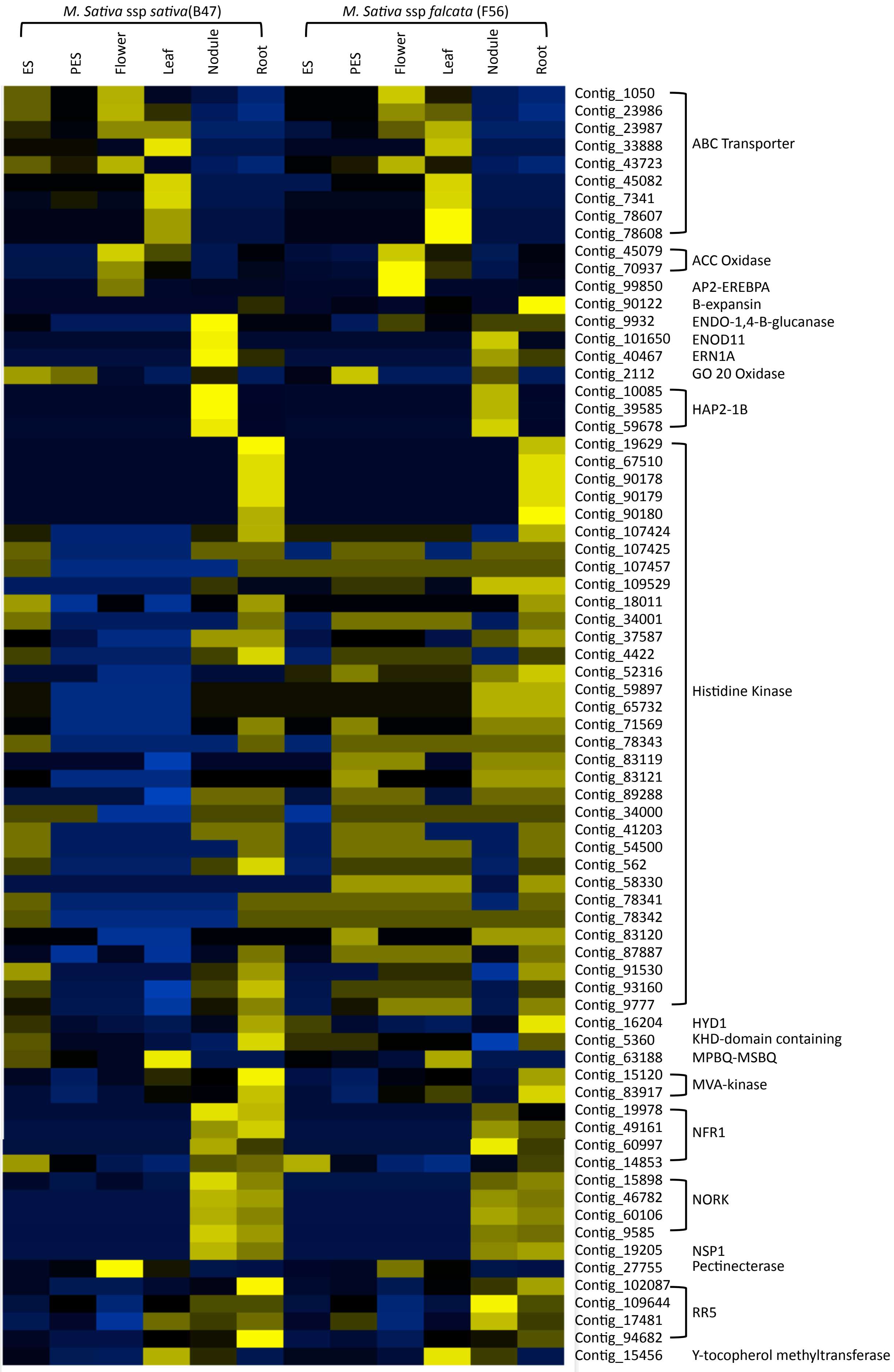

Expression profile of transcripts likely involved in nodulation. Expression presented as Z-scores: yellow, above average expression; blue, below average expression. ES, elongating stem internodes; PES, post-elongating stem internodes.
